# Neighborhood income and major depressive disorder in a large Dutch population: results from the LifeLines Cohort study

**DOI:** 10.1186/s12889-016-3332-2

**Published:** 2016-08-11

**Authors:** Bart Klijs, Eva U. B. Kibele, Lea Ellwardt, Marij Zuidersma, Ronald P. Stolk, Rafael P. M. Wittek, Carlos M. Mendes de Leon, Nynke Smidt

**Affiliations:** 1 Department of Epidemiology, University of Groningen, University Medical Center Groningen, P.O. Box 30001, 9700 RB Groningen, The Netherlands; 2 Population Research Centre, Faculty of Spatial Sciences, University of Groningen, P.O. Box 800, 9700 AV Groningen, The Netherlands; 3 Institute of Sociology and Social Psychology, University of Cologne, Albert-Magnus-Platz 50923, Cologne, Germany; 4 Department of Psychiatry, Interdisciplinary Center Psychopathology and Emotion regulation, University of Groningen, University Medical Center Groningen, P.O. Box 30001, 9700 RB Groningen, The Netherlands; 5 Department of Epidemiology, University of Michigan School of Public Health, 1415 Washington Heights, Ann Arbor, 48109-2029 MI USA

**Keywords:** Depressive disorder, Income, Psychosocial deprivation, Residence characteristics, Socioeconomic factors

## Abstract

**Background:**

Previous studies are inconclusive on whether poor socioeconomic conditions in the neighborhood are associated with major depressive disorder. Furthermore, conceptual models that relate neighborhood conditions to depressive disorder have not been evaluated using empirical data. In this study, we investigated whether neighborhood income is associated with major depressive episodes. We evaluated three conceptual models. Conceptual model 1: The association between neighborhood income and major depressive episodes is explained by diseases, lifestyle factors, stress and social participation. Conceptual model 2: A low individual income relative to the mean income in the neighborhood is associated with major depressive episodes. Conceptual model 3: A high income of the neighborhood buffers the effect of a low individual income on major depressive disorder.

**Methods:**

We used adult baseline data from the LifeLines Cohort Study (*N* = 71,058) linked with data on the participants’ neighborhoods from Statistics Netherlands. The current presence of a major depressive episode was assessed using the MINI neuropsychiatric interview. The association between neighborhood income and major depressive episodes was assessed using a mixed effect logistic regression model adjusted for age, sex, marital status, education and individual (equalized) income. This regression model was sequentially adjusted for lifestyle factors, chronic diseases, stress, and social participation to evaluate conceptual model 1. To evaluate conceptual models 2 and 3, an interaction term for neighborhood income*individual income was included.

**Results:**

Multivariate regression analysis showed that a low neighborhood income is associated with major depressive episodes (OR (95 % CI): 0.82 (0.73;0.93)). Adjustment for diseases, lifestyle factors, stress, and social participation attenuated this association (ORs (95 % CI): 0.90 (0.79;1.01)). Low individual income was also associated with major depressive episodes (OR (95 % CI): 0.72 (0.68;0.76)). The interaction of individual income*neighborhood income on major depressive episodes was not significant (*p* = 0.173).

**Conclusions:**

Living in a low-income neighborhood is associated with major depressive episodes. Our results suggest that this association is partly explained by chronic diseases, lifestyle factors, stress and poor social participation, and thereby partly confirm conceptual model 1. Our results do not support conceptual model 2 and 3.

## Background

Major depressive disorder is a mental disorder that is characterized by a severely depressed mood during most of the day, nearly every day, and a loss of interest in almost all activities [1]. Major depressive disorder is common in the general population and is a burden for the individual [2]. In the Global Burden of Disease Study, major depressive disorder is ranked fifth on the list of conditions associated with the largest burden of disease [2]. Various personal factors such as genes, adverse life events, personality traits, and somatic diseases are associated with major depressive episodes [3–6]. Furthermore, also factors in peoples’ environment, such as the neighborhood in which people live, can influence the risk of major depressive episodes [7].

Studies investigating the role of the neighborhood environment in the development of major depressive disorder have produced inconsistent results on whether living in a neighborhood of poorer socioeconomic conditions is associated with major depressive episodes [7–17]. Studies in New York City, Chicago and the metropolitan area of Paris have found that persons living in neighborhoods of lower socioeconomic status have a higher risk of depressive symptoms [7, 10, 11]. Other studies, however, have either failed to find a significant relationship between neighborhood socioeconomic conditions and mental health, or found that this association is fully explained by the socioeconomic position of individuals [13–17].

Various conceptual models have been proposed to explain the potential link between neighborhood conditions and major depressive episodes. In a systematic review, Kim proposed a model of absolute poverty in which unfavorable material and psychosocial conditions are concentrated in less affluent neighborhoods [18]. These unfavorable conditions are associated with the presence of chronic diseases, an unhealthy lifestyle, increased stress and lower social participation. In turn, each of these factors may give rise to episodes of major depression. Drawing on social comparison theory, others have proposed a model of relative poverty to explain the link between neighborhood conditions and major depressive episodes [19–22]. It is suggested that a low income relative to others increases the probability of negative self-evaluations and causes psychosocial stress and depression in the long run. Within the context of the neighborhood, this would mean that a low income is particularly problematic for individuals living in high-income neighborhoods. In contrast, the collective resources model suggests a beneficial effect of living in a high-income neighborhood for those with a low income [12, 23]. This model proposes that services, facilities and social capital are more widely available in rich than in poor areas. Individuals with a low income, who may be less in the position to purchase goods and services privately, may benefit from the collective resources in a high-income neighborhood.

The various conceptual models proposed in the literature have rarely been evaluated on the basis of empirical data. Only one study has evaluated the collective resources and relative poverty models in a neighborhood context [12]. This study favored the collective resources model over the relative poverty model, but lacked power to draw definitive conclusions. Our aim is to investigate to what extent neighborhood income is associated with major depressive episodes. We evaluate three conceptual models linking neighborhood income to major depressive episodes. Conceptual model 1: The association between neighborhood income and major depressive episodes is explained by diseases, lifestyle factors, stress and social participation. Conceptual model 2: A low individual income relative to the mean income in the neighborhood is associated with major depressive episodes. Conceptual model 3: A high income of the neighborhood buffers the effect of a low individual income on major depressive disorder.

## Methods

### Study population

We used a first released baseline subsample of the Dutch LifeLines Cohort Study that included 71,514 participants who had been recruited between 2006 and 2012 [24–26]. The cohort profile of LifeLines is described elsewhere [25]. Briefly, LifeLines is a large representative population based cohort study aiming to investigate universal risk factors for multifactorial diseases that has been shown to be broadly representative for the adult population in the northern part of the Netherlands [26]. The recruitment of participants (*N* = 167,729) was carried out between 2006 and 2013. Informed consent was obtained from all individual participants included in the study. All participants visited one of the LifeLines research sites, where anthropometric and blood pressure measurements were taken and fasting (12 hours) blood samples were collected. Participants filled out extensive questionnaires including items on demographic and socioeconomic characteristics, chronic diseases, health behaviors, stress and social participation. The participants’ home addresses were geo-coded and linked with information on the neighborhood available through Statistics Netherlands [27]. We excluded 456 individuals (0.6 %) with missing information on major depressive episodes, which resulted in a study sample of 71,058 individuals.

### Current episode of major depressive disorder

The Mini International Neuropsychiatric Interview (MINI) was used to assess the presence of a current (in past two weeks) major depressive episode according to standard criteria in the Diagnostic Statistical Manual (DSM)-IV [28]. The MINI is a validated and reliable brief structured oral interview for the major Axis I psychiatric disorders in DSM-IV and ICD-10, including major depressive episodes [28]. The interview assesses two core symptoms (consistently depressed or down; and much less interested in most things) and seven related symptoms (loss of appetite; trouble sleeping; talking or moving more slowly/restlessness; tiredness; feelings of worthlessness and guilt; difficulty concentrating or making decisions; and considering to hurt yourself/suicidal). A major depressive episode is established when a person has at least one main symptom and at least five symptoms in total. Interviews were administered by trained research assistants.

### Neighborhood income and individual equalized income

Neighborhood income was defined as the mean disposable income in the neighborhood of individuals with an income during the entire year, for the year 2009. This information was available from Statistics Netherlands and was downloaded from the website (www.cbs.nl/nl-nl/dossier/nederland-regionaal/wijk-en-buurtstatistieken). The variable for neighborhood income was continuous with one point increase indicating 500 €/month higher income.

LifeLines participants were asked to report their net household income according to eight categories (less than 750; 750–1000; 1000–1500; 1500–2000; 2000–2500; 2500–3000; 3000–3500; more than 3500 €/month). Furthermore, they were asked how many people live on this amount. The individual income was equalized according to the square root scale method, according to which the net household income is divided by square root of the number of persons living on this amount [29]. For this calculation, middle values of the income categories were used (€500 and €3750 for the outer categories). The resulting variable was used as a continuous indicator with one point increase indicating 500 €/month higher income. In our data, the Pearson’s correlation coefficient for neighborhood income and individual income was 0.17 (*P* < 0.001).

### Chronic diseases and life style factors

A variety of diseases are associated with major depressive disorder [6]. In the questionnaire, study participants were asked to report on the current presence of osteoarthritis, rheumatoid arthritis, chronic obstructive pulmonary disease, diabetes mellitus, myocardial infarction, heart failure, cerebrovascular accident, Crohn’s disease, hepatitis, liver cirrhosis, multiple sclerosis, Parkinson’s disease, dementia and psoriasis was assessed. A variable indicating the number of these diseases present (none, one, two or more) was used in the analysis.

Body mass index (BMI) was calculated using measured body weight and length (BMI = weight/length^2^) and was categorized into ‘underweight’ (<18.5 kg/m^2^), ‘normal weight’ (18.5–24.9 kg/m^2^), ‘overweight’ (25–29.9 kg/m^2^), and ‘obesity class I’ (30–35 kg/m^2^) and ‘obesity class II’ (> = 35 kg/m^2^). Using two questions asking “Have you ever smoked for a full year?” and “Do you currently smoke, or have you smoked during the past month?”, smoking behavior was categorized as ‘never’, ‘past’ and ‘current’ smoker. Study participants were asked to report the number of days per week on which they were active (i.e. cycling, gardening, doing odd jobs or sports activities) for at least half an hour. The answers were categorized into none, one, two, and three or more days per week. Alcohol consumption was assessed using two questions asking “How often (on how many days) did you consume alcohol during the past month?” and “On a day of drinking, how many alcoholic beverages did you take on average?”. Using these questions, alcohol consumption was categorized into ‘abstainer’, ‘moderately drinking’ (<=1 glass daily on average for females; <=2 glasses daily on average for males) and ‘heavily drinking’ (>1 glass daily on average for females; >2 glasses daily on average for males).

### Acute and long-term stress

Acute and chronic stressors were measured using the List of Threatening Events (LTE) and the Long-term Difficulties Inventory (LDI) [30, 31]. The LTE measures the occurrence of 12 life events with established long-term consequences in the past year, such as the death of a close friend or relative. A continuous variable indicating the number of events in the past year (range 0–12) was used. The LDI consisted of 12 items evaluating to what extent various domains of life including housing, work, social relationships, free time, finances, health, school/study, and religion had been perceived as stressful during the last year. Respondents indicated how they experienced these aspects on a three-point scale (0 = not stressful, 1 = slightly stressful, 2 = very stressful). A sum score was calculated (range 0–24) with higher scores indicating higher stress from long-term difficulties. The test-retest correlations of the LTE and LDI in a two-year interval are 0.61 and 0.72. LDI scores have been shown to correlate with psychological distress (*r* = 0.42) and neuroticism (*r* = 0.39) [31]. In our data, Pearson’s correlation coefficient for acute and long-term stress was 0.32 (*P* < 0.001).

### Social participation

The size of the social network was assessed as the average number of personal contacts in which personal matters were exchanged or discussed, either through written or oral communication, within a period of two weeks. A continuous variable was created with one point increase indicating five more personal contacts. To account for a non-linear relationship between the number of personal contacts and major depressive episodes, also a quadratic term for the number of personal contacts was used. Participation in organized clubs and groups was measured using a continuous variable (range 0–6) indicating the number of participations in sports clubs, neighborhood or social clubs, political parties, patient associations, church or religious communities, and other clubs. The quality of social contacts was assessed using nine items from the Social Production Function Instrument for Level of Well-Being (SPF-IL) measuring ‘affection’, ‘behavioral confirmation’ and ´status´ [32]. Answers could be given on a four-point scale ranging from never (0) to always (3). A sum score was calculated (range 0–27) with higher scores indicating higher social need fulfillment. The test-retest correlations of affection, behavioral confirmation and status are between 0.6 and 0.7 [31]. The SPF-IL score correlates with traditional measures of well-being (*r* = 0.6) [32].

### Other control variables

Level of education was categorized as ‘tertiary’, (community or junior college, vocational technical institute, university), ‘upper secondary’ (senior general secondary, pre-university), ‘lower secondary’ (junior general secondary, senior secondary vocational) and ‘elementary’ (no education, elementary, prevocational, lower vocational). Age (in years) was measured on a continuous scale. Dichotomous variables were created for sex (male/female) and marital status (married or registered partnership; yes/no).

### Statistical analysis

Characteristics of the study participants and the study participants’ neighborhoods were presented for all residential areas and according to neighborhood income. Univariate and multivariate mixed effect logistic regression models were used to assess the association between neighborhood income, individual income and major depressive episodes. A random intercept was included to account for clustered observations within neighborhoods. To evaluate the conceptual model of absolute poverty, a multivariate model was fitted with neighborhood income as main independent variable and major depressive episode as dependent variable (regression model 1). The model was adjusted for age, sex, marital status, individual income and education. This model was sequentially adjusted for chronic diseases and lifestyle factors (regression model 2), stress (regression model 3) and social participation (regression model 4), which explain the association between neighborhood income and major depressive episodes according to the absolute poverty model. The conceptual models of relative poverty and collective resources both assume an interactive effect of individual income and neighborhood income to the prevalence of major depressive episodes. According to the relative poverty model, the effect of lower individual income on major depressive episodes is stronger in neighborhoods of higher income. In a regression model this would yield a significant negative interaction effect of individual income*neighborhood income. According to the collective resources model, the effect of lower individual income is weaker in neighborhoods of higher income, which would yield a significant positive interaction of individual income*neighborhood income. To evaluate the conceptual models of relative poverty and collective resources, an interaction term of individual income*neighborhood income was added to model 1 (regression model 5). For all models, the standard deviation of the random intercept for neighborhood was presented. Merlo et al. recommend the median odds ratio as an indicator of the group level (neighborhood) variance in mixed effect logistic regression models [33]. However, the Stata function to calculate the median odds ratio (xtmrho) was incompatible with the multiple imputation procedure followed. Therefore, median odds ratios were only presented for the complete case analyses. Using model 1, the prevalence of major depressive episodes was estimated by neighborhood income and individual income (1^st^ and 9^th^ deciles). Missing values of all independent variables were imputed using multiple imputations, using the multivariate normal model function (mi impute mvn) of Stata. We used a multivariate normal model in which age, sex, major depressive episodes, number of chronic diseases, number of personal contacts, participation in clubs or groups and neighborhood income were the predictor variables. The percentage of missing values for each variable are shown in Table 1.

**Table 1:** Characteristics of study population

		Neighborhood income			
	All residential areas	Less than 1400 €/month	1400-1599 €/month	1600-1799 €/month	1800 €/month or more
N	71058	9315	29970	18400	13373
% Current major depressive episode	2.5	3.3	2.8	2.3	1.6
Individual equivalized income					
Mean, sd	1514 (568)	1388 (556)	1455 (554)	1555 (570)	1673 (565)
% Missing	15.1	16.3	15.9	14.3	13.7
Demographic characteristics					
Age (mean, sd)	43.7 (11.6)	42.1 (11.9)	43.3 (11.6)	44.1 (11.5)	45.4 (11.3)
% Female	58.0	59.0	58.1	57.8	57.3
% Married	61.6	53.9	61.5	62.4	66.2
Highest education					
% Elementary	2.2	3.1	2.6	1.6	1.5
% Lower secondary	26.1	30.3	29.3	23.5	19.4
% Upper secondary	39.7	40.7	41.1	40.0	35.4
% Tertiary	29.9	23.8	24.9	32.7	41.4
% Missing	2.2	2.2	2.2	2.2	2.3
Diseases					
% No disease	77.0	75.4	76.0	77.9	79.0
% One disease	9.5	9.8	9.9	8.9	9.2
% Two or more diseases	13.5	14.9	14.1	13.2	11.8
Body mass index					
% < =18.5 kg/m2	0.8	0.8	0.8	0.8	0.7
% 18.5 - 24.9 kg/m2	45.0	44.6	42.8	45.6	49.1
% 25–29.9 kg/m2	38.8	36.9	39.2	39.4	38.3
% 30.0 - 34.9 kg/m2	11.5	12.6	12.6	10.7	9.5
% > =35 kg/m2	3.9	5.0	4.5	3.5	2.5
Physical activity (at least 30. min)					
% Physically inactive	4.3	5.3	4.7	3.9	3.4
% One day per week	8.2	8.4	8.8	8.2	7.0
% Two days per week	11.2	11.5	11.4	11.4	10.5
% Three or more days per week	70.3	67.9	68.6	71.4	74.4
Smoking					
% Never smoking	43.3	42.3	41.4	45.1	45.8
% Formerly smoking	28.8	26.8	28.2	29.2	31.0
% Currently smoking	22.3	26.1	24.0	20.8	18.0
% Missing	5.6	4.8	6.4	4.9	5.2
Alcohol consumption^a^					
% Abstaining	21.2	23.2	23.5	19.5	17.0
% Moderately drinking	61.4	60.4	58.5	63.7	65.3
% Heavily drinking	14.4	14.2	14.7	14.0	14.6
% Missing	3.0	2.2	3.2	2.9	3.1
Stress					
No. of threatening events in past year (LTE), 0–12 (mean, sd)	1.1 (1.3)	1.2 (1.4)	1.1 (1.3)	1.0 (1.3)	1.0 (1.2)
% Missing	2.6	3.2	2.7	2.2	2.3
Score on long-term difficulty inventory (LDI), 0–24 (mean, sd)	2.4 (2.4)	2.7 (2.5)	2.4 (2.4)	2.4 (2.3)	2.3 (2.3)
% Missing	3.3	4.1	3.3	2.9	3.1
Social participation					
No. of personal contacts in past two weeks (mean, sd)	18.2 (14.5)	17.8 (14.6)	18.1 (14.5)	18.3 (14.4)	18.8 (14.6)
No. of participations in clubs or groups, 0–6 (mean, sd)	0.9 (0.9)	0.9 (0.9)	0.9 (0.9)	1.0 (0.9)	0.9 (0.9)
Score on social need fulfillment scale (SPF-IL), 0–27 (mean, sd)	16.0 (3.5)	15.7 (3.6)	15.9 (3.6)	16.0 (3.4)	16.3 (3.4)
% Missing	3.7	4.4	3.9	3.4	3.2

^a^Heavily drinking is > = 14 (men) or > =7 alcoholic consumptions per week

In the northern part of the Netherlands, a large part of the individuals aged 18–30 are students, who generally have a low income but a prospect of a high socioeconomic position. We performed a sensitivity analysis in which we evaluated to what extent the associations changed when individuals aged 18–30 years were excluded. A complete case analysis was performed to evaluate the potential impact of the imputation procedure on our substantive conclusions. Furthermore, we evaluated to what extent our results changed when ‘percentage of low income households’ (Z-standardized; disposable household income < 25,100 euro, set by Statistics Netherlands) instead of ‘mean income’ was used as in indicator of neighborhood poverty.

## Results

Our study population consisted of 71,058 individuals with a mean age of 43.7 years (sd 11.6). Of the participants, 58.0 % were female and 61.6 % were married or had a registered partnership. The prevalence of major depressive episodes in our study population was 2.5 % and varied from 1.6 % in the high-income neighborhoods to 3.3 % in the low-income neighborhoods. In general, persons living in a low-income neighborhood were slightly younger, had a lower education, were more often married or had a registered partnership, had more diseases, an unhealthier lifestyle, more stressful life events and more long-term difficulties than persons living in a high-income neighborhood. Details of the background characteristics of our study population are presented in Table 1.

Our study participants resided in 1893 different neighborhoods. The mean number of participants per neighborhood was 37.5 (range 1 to 1001). As compared with high-income neighborhoods, low-income neighborhoods were less frequently located in a strongly urbanized area with more than1500 addresses/km^2^ (25.6 % versus 32.6 %) and had a higher percentage of non-western migrants (9.6 % versus 5.1 %). The difference in the percentage of single occupied houses and residents older than 65 years was smaller than 3 %. Low-income neighborhoods had a smaller percentage of owner occupied houses, and a larger percentage of households receiving assistance benefits and households living below social minimum than high-income neighborhoods (all differences 4.8 % or more). Characteristics of the study participants’ neighborhoods are presented in Table 2.

**Table 2:** Characteristics of study participants’ neighborhoods

		Neighborhood income			
	All residential areas	Less than 1400 €/month	1400-1599 €/month	1600-1799 €/month	1800 €/month or more
N	1893	289	552	484	568
Demographic characteristics					
Neighborhoods in strongly urbanized area (N, %)^a^	456 (24.1)	74 (25.6)	104 (18.8)	93 (19.2)	185 (32.6)
Non-western migrants (%, sd)	5.5 (8.7)	9.6 (14.6)	4.8 (7.9)	4.3 (6.5)	5.1 (6.1)
Single occupied households (%, sd)	31.2 (15.2)	33.8 (17.5)	31.3 (13.8)	29.9 (13.7)	30.9 (16.2)
Population older than 65 years (%, sd)	15.1 (8.0)	13.4 (6.2)	15.1 (7.3)	15.1 (7.6)	15.8 (9.5)
Socioeconomic characteristics					
Owner occupied houses (%, sd)	64.9 (20.6)	55.6 (26.4)	62.7 (19.2)	67.3 (16.8)	69.8 (19.5)
Households receiving assistance benefits (%, sd)	3.8 (3.5)	7.0 (5.6)	4.3 (3.2)	2.9 (2.0)	2.2 (1.9)
Households living below social minimum (%, sd)^b^	8.1 (5.1)	12.9 (6.6)	8.7 (4.5)	7.1 (3.8)	6.1 (3.8)

^a^More than 1500 addresses per km^2^. ^b^Disposable household income less than 25100 per year

Table 3 presents the results of the univariate and multivariate logistic regression analyses on major depressive episodes. In the univariate analyses, all variables were significantly associated with major depressive episodes, except for the interaction between neighborhood income and individual income. In the multivariate regression model adjusted for age, sex, marital status and education (regression model 1), higher neighborhood income (OR with 95 % CI is 0.82 (0.73;0.93)) and higher individual income (OR with 95 % CI is 0.72 (0.68;0.76)) were associated with major depressive episodes. Sequential adjustment for lifestyle and diseases (regression model 2), short-term and long-term stress (regression model 3), and social participation (regression model 4) attenuated the associations of neighborhood income and individual income with major depressive episodes (ORs with 95 % CI are 0.90 (0.97;1.01) for neighborhood income and 0.92 (0.87-0.98) for individual income). There was no significant interaction effect of neighborhood income*individual income to the prevalence of major depressive episodes (regression model 5). In regression model 4, low education, presence of chronic diseases, high body mass index, physical inactivity, current smoking, alcohol consumption, threatening events, long term difficulties, fewer personal contacts, few participations in organized clubs or groups and low social need fulfillment were all independently associated with major depressive episodes.

**Table 3:** Univariate and multivariate mixed effect logistic regression models on prevalence of current major depressive disorder

		Evaluation of conceptual model of absolute poverty				Evaluation of conceptual models of relative poverty and collective resources
	Univariate logistic regression models^e^	Regression model 1	Regression model 2	Regression model 3	Regression model 4	Regression model 5
	(OR with 95 % CI)	(OR with 95 % CI)	(OR with 95 % CI)	(OR with 95 % CI)	(OR with 95 % CI)	(OR with 95 % CI)
Neighborhood and household income						
Neighborhood income^a^	0.64 (0.57;0.73)	0.82 (0.73;0.93)	0.89 (0.79;1.00)	0.87 (0.77;0.98)	0.90 (0.79;1.01)	0.69 (0.52;0.92)
Individual equalized income^a^	0.62 (0.59;0.65)	0.72 (0.68;0.76)	0.75 (0.71;0.80)	0.89 (0.83;0.95)	0.92 (0.87;0.98)	0.59 (0.43;0.80)
Neighborhood income*individual equalized income	1.06 (0.96;1.18)^d^					1.07 (0.97;1.17)
Demographic characteristics						
Age	0.99 (0.99;1.00)	1.00 (1.00;1.00)	1.00 (0.99;1.00)	1.01 (1.01;1.02)	1.01 (1.00;1.01)	1.00 (1.00;1.00)
Female	1.62 (1.46;1.79)	1.46 (1.32;1.62)	1.38 (1.23;1.54)	1.24 (1.10;1.39)	1.27 (1.13;1.42)	1.46 (1.31;1.62)
Married	0.53 (0.48;0.59)	0.55 (0.49;0.61)	0.58 (0.52;0.65)	0.79 (0.71;0.88)	0.80 (0.72;0.90)	0.55 (0.49;0.61)
Highest education						
Tertiary	Ref.	Ref.	Ref.	Ref.		Ref.
Upper secondary	1.80 (1.55;2.08)	1.42 (1.21;1.65)	1.28 (1.09;1.50)	1.50 (1.28;1.76)	1.37 (1.17;1.61)	1.42 (1.22;1.66)
Lower secondary	3.07 (2.65;3.55)	2.44 (2.07;2.87)	1.96 (1.66;2.32)	2.58 (2.17;3.06)	2.10 (1.76;2.50)	2.45 (2.07;2.88)
Elementary	5.98 (4.76;7.51)	4.03 (3.15;5.16)	3.02 (2.35;3.90)	3.99 (3.05;5.22)	2.97 (2.26;3.90)	4.05 (3.16;5.18)
Diseases						
No disease	Ref.		Ref.	Ref.		
One disease	1.88 (1.63;2.16)		1.61 (1.39;1.86)	1.43 (1.23;1.66)	1.43 (1.22;1.66)	
Two or more diseases	2.23 (1.99;2.51)		1.84 (1.63;2.07)	1.42 (1.26;1.61)	1.36 (1.20;1.55)	
Body mass index						
<=18.5 kg/m2	1.98 (1.32;2.99)		1.36 (0.89;2.06)	1.39 (0.89;2.15)	1.31 (0.84;2.04)	
18.5–24.9 kg/m2	Ref.		Ref.	Ref.	Ref.	
25–29.9 kg/m2	0.92 (0.82;1.03)		0.95 (0.85;1.07)	0.91 (0.80;1.02)	0.92 (0.82;1.04)	
30.0–34.9 kg/m2	1.52 (1.32;1.75)		1.30 (1.12;1.50)	1.21 (1.04;1.41)	1.20 (1.03;1.40)	
> = 35 kg/m2	2.82 (2.37;3.34)		1.89 (1.58;2.25)	1.57 (1.30;1.90)	1.51 (1.25;1.84)	
Physical activity (at least 30. minutes)						
Three or more days per week	Ref.		Ref.	Ref.		
Two days per week	1.06 (0.90;1.24)		1.01 (0.85;1.21)	1.03 (0.86;1.24)	1.00 (0.84;1.21)	
One day per week	1.48 (1.27;1.74)		1.26 (1.07;1.47)	1.26 (1.06;1.49)	1.16 (0.97;1.38)	
Physically inactive	2.22 (1.86;2.65)		1.57 (1.28;1.92)	1.58 (1.28;1.95)	1.42 (1.15;1.75)	
Smoking						
Never smoking	Ref.		Ref.	Ref.		
Formerly smoking	1.01 (0.89;1.14)		1.07 (0.93;1.22)	0.99 (0.87;1.14)	1.00 (0.87;1.14)	
Currently smoking	2.12 (1.89;2.39)		1.76 (1.55;2.00)	1.45 (1.27;1.65)	1.46 (1.28;1.67)	
Alcohol consumption^b^						
Abstaining	2.11 (1.89;2.35)		1.65 (1.47;1.84)	1.70 (1.51;1.91)	1.52 (1.34;1.71)	
Moderately drinking	Ref.		Ref.	Ref.	Ref.	
Heavily drinking	1.21 (1.04;1.40)		1.17 (1.00;1.36)	1.22 (1.04;1.43)	1.19 (1.01;1.40)	
Stress						
No. of threatening events in past year (LTE)	1.54 (1.50;1.57)			1.18 (1.15;1.22)	1.19 (1.16;1.23)	
Score on long-term difficulty inventory (LDI)	1.38 (1.36;1.40)			1.32 (1.30;1.34)	1.27 (1.24;1.29)	
Social participation						
No. of personal contacts in past two weeks	0.64 (0.60;0.69)				0.85 (0.79;0.91)	
No. of personal contacts in past two weeks squared ^c^	1.04 (1.03;1.04)				1.02 (1.01;1.02)	
No. of participations in clubs or groups	0.65 (0.61;0.69)				0.87 (0.86;0.89)	
Score on social need fulfillment scale (SPF-IL)	0.80 (0.79;0.81)				0.83 (0.77;0.88)	
sd random intercept neighborhood		0.32 (0.24;0.41)	0.28 (0.21;0.38)	0.27 (0.19;0.38)	0.24 (0.16;0.37)	0.31 (0.24;0.41)

^a^Continuous variables in which one point equals 500 €/month. ^b^Heavily drinking is > = 14 (men) or > =7 alcoholic consumptions per week. ^c^A quadratic term for personal contacts was included because of a non-linear relationship with depression. ^d^Interaction effect adjusted for neighborhood income and individual equalized income. ^e^All univariate logistic regression models include a random intercept for neighborhood

**Fig. 1:**
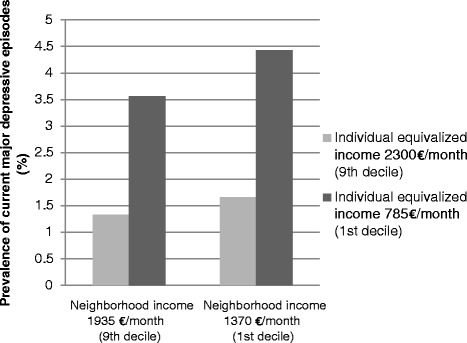
Prevalence of major depressive episodes by neighborhood income and individual equivalized income

Figure 1 presents the prevalence of major depressive episodes by neighborhood income and individual income using the coefficients estimated in model 1. The figure shows that the prevalence of major depressive episodes differs by neighborhood income and by individual income, but more so by individual income. Among persons with a low individual income, the prevalence of major depressive episodes is 0.9 percentage points higher for those living in a low-income neighborhood (4.4 %) than for those in a high-income neighborhood (3.6 %). The prevalence of major depressive episodes among persons in low-income neighborhoods is 2.8 percentage points higher for persons with a low individual income (4.4 %) than for those with a high individual income (1.7 %).

### Sensitivity analysis

Using the percentage of low-income households instead of the mean income in the neighborhood as an indicator of neighborhood poverty did not affect our results and conclusions. Also using complete cases only (*N* = 50,288) instead of imputed data, or excluding persons younger than 30 from our dataset did not alter our results (Table 4).

**Table 4:** Multivariate mixed logistic regression models using an alternative indicator of neighborhood poverty, and for complete cases and individuals older than 30 years

	Evaluation of conceptual model of absolute poverty				Evaluation of conceptual models of relative poverty and collective resources
	Regression model 1	Regression model 2	Regression model 3	Regression model 4	Regression model 5
	(OR with 95 % CI)	(OR with 95 % CI)	(OR with 95 % CI)	(OR with 95 % CI)	(OR with 95 % CI)
Alternative indicator of neighborhood poverty (*N* = 70982)					
Percentage low income households in neighborhood (Z-standardized)	1.16 (1.09;1.23)	1.12 (1.06;1.19)	1.09 (1.03;1.15)	1.08 (1.02;1.14)	1.24 (1.09;1.42)
Individual equalized income	0.72 (0.68;0.77)	0.75 (0.70;0.79)	0.88 (0.83;0.94)	0.91 (0.86;0.97)	0.78 (0.67”0.92)
Percentage low income households in neighborhood*individual equalized income					0.98 (0.93;1.02)
sd random intercept neighborhood	0.30 (0.23;0.40)	0.25 (0.17;0.36)	0.23 (0.14;0.36)	0.23 (0.14;0.37)	0.30 (0.23;0.39)
Complete cases (*N* = 50288)					
Neighborhood income	0.82 (0.71;0.94)	0.89 (0.78;1.02)	0.90 (0.79;1.04)	0.93 (0.81;1.06)	0.65 (0.46;0.91)
Individual equalized income	0.75 (0.71;0.80)	0.77 (0.73;0.82)	0.92 (0.86;0.98)	0.95 (0.89;1.02)	0.58 (0.40;0.82)
Neighborhood income*individual equalized income					1.09 (0.97;1.21)
sd random intercept neighborhood	0.29 (0.21;0.39)	0.24 (0.16;0.39)	0.22 (0.13;0.37)	0.21 (0.12’0.38)	0.28 (0.21;0.39)
Individuals older than 30 years (*N* = 61463)					
Neighborhood income	0.84 (0.74;0.96)	0.90 (0.79;1.02)	0.89 (0.79;1.01)	0.91 (0.80;1.03)	0.74 (0.53;1.04)
Individual equalized income	0.70 (0.65;0.76)	0.73 (0.68;0.78)	0.87 (0.81;0.94)	0.91 (0.85;0.98)	0.61 (0.42;0.87)
Neighborhood income*individual equalized income					1.05 (0.94;1.17)
sd random intercept neighborhood	0.07 (0.03;0.18)	0.02 (0.00;0.27)	0.02 (0.00;0.40)	0.02 (0.00;0.49)	0.07 (0.03;0.18)
Median odds ratio (MOR)	1.28	1.15	1.15	1.14	1.28

## Discussion

Our aim was to investigate whether neighborhood income is associated with major depressive episodes. We evaluated three conceptual models linking neighborhood income to major depressive episodes. We found that living in a low-income neighborhood is associated with major depressive episodes. Our results partly confirm the model of absolute poverty, but suggest that besides chronic diseases, lifestyle factors, stress and social participation, other factors explain the link between neighborhood income and major depressive episodes [18]. We did not find an interactive effect of neighborhood income and individual income to major depressive episodes. This means that our results do not support the conceptual models of relative poverty and collective resources.

Our study is one of the first studies showing that persons in low-income neighborhoods suffer more often from major depressive episodes than persons in high-income neighborhoods. Three previous studies have found a relationship between some indicator of socioeconomic conditions in the neighborhood and depressive symptomatology [7, 10, 11]. However, only one of these studies assessed depressive symptoms in relation to neighborhood income [7]. The study sample in this study (*N* = 7290), by Annequin et al., was ten times smaller than our study sample. Furthermore, the study was restricted to neighborhoods from the highly urbanized agglomeration of Paris, and self-report instead of face-to-face interviews, as in our study, was used to assess the presence of depressive symptoms [7]. Annequin et al. found that higher neighborhood income is associated with a lower prevalence of depressive symptoms, which is in line with our results [7]. In contrast with our study, they did not evaluate which individual factors explain this association [7]. In our analysis, the association between neighborhood income and major depressive episodes is only partly explained by diseases, lifestyle factors, stress and social participation, which suggests that Kim’s conceptual model of absolute poverty is insufficient to fully explain the differential distribution of major depressive episodes by neighborhood income [18]. In addition to the factors included in Kim’s model, studies have shown that personality traits, childhood experiences, and genetic factors play a role in the development of major depressive episodes [3–5]. Furthermore, few facilities, few parks, poor walkability, residential instability, and social fragmentation in the neighborhood are associated with determinants of major depressive disorder such as stress and physical inactivity [34–39]. A differential distribution by neighborhood income of each of these factors can further explain the association between neighborhood income and major depressive episodes.

### Evaluation of data and methods

The prevalence of major depressive episodes in our study (2.5 %) is lower than in most other studies (5 to 10 %). This difference can be explained by the fact that we assessed the presence of major depressive episodes in the past two weeks, whereas other studies assessed the one-year prevalence [40, 41]. Strengths of our study include the large study population of 71,058 participants from almost 1900 neighborhoods in both rural and urban areas. Previous studies often evaluated socioeconomic conditions on a less detailed geographical level, such as income inequality at the country or state level [19, 42]. The presence of a major depressive episode was evaluated using a validated diagnostic interview, while many previous studies relied on self-report questionnaires [9, 10, 16]. Our study also has some limitations. First, socioeconomic status was operationalized solely based on income, as alternative indicators such as educational attainment were not available on the neighborhood level. Second, exposure to a certain neighborhood is typically long, whereas the measure of major depressive episodes covered a comparatively short time-span of two weeks only. Because of this, the effect of neighborhood socioeconomic status on severe and chronic forms of depression may have been underestimated. Third, the results produced in this research are based on cross-sectional data and thus, strictly speaking, do not allow causal inferences. A low individual income can be a cause as well as a consequence of major depressive episodes (reverse causation). Perhaps some of the study participants suffered from depression prior to moving to their neighborhood, or depression withheld them from moving to a neighborhood of higher income. Experiencing economic hardship can be a result of poor mental health and force individuals to seek for affordable places to live, i.e. in relatively poor areas. When investigating effects of relative poverty, a particular challenge is the selection of the appropriate reference group. In our study we used the mean income level in the neighborhood as a reference and found no evidence supporting an effect of relative poverty. However, people may compare themselves with individuals from a more restricted group in the same neighborhood or to individuals from the general population.

## Conclusions

People living in low-income neighborhoods or with a low individual income more often suffer from major depressive episodes. This higher risk is partly explained by the presence of diseases, a less healthy lifestyle, stress and a lack of social participation. A low income relative to the mean income in the neighborhood is not associated with major depressive episodes. Living in a high-income neighborhood does not buffer the effect of a low individual income on major depressive episodes. Evidence from randomized controlled trials suggests that lifestyle programs combining behavior modification, physical activity and adjustment of diet; stress reduction programs; and social interventions can contribute to a healthier lifestyle, fewer stress, and higher social participation [43–45]. Targeting these interventions at the group at risk for developing major depressive episodes, i.e. persons who live in a low-income neighborhood and also have a low individual income, can be an efficient way to reduce the prevalence of major depressive episodes in the population.

## Abbreviations

BMI, body mass index; DSM-IV, diagnostic and statistical manual of mental disorders, 4th edition; ICD-10, International Statistical Classification of Diseases and Related Health Problems, 10^th^ edition; km, kilometers; LDI, long-term difficulties inventory; LTE, list of threatening events; MINI, Mini International Neuropsychiatric Interview
